# A two-year comparative assessment of retention of arch width increases between modified vacuum-formed and Hawley retainers: a multi-center randomized clinical trial

**DOI:** 10.1186/s40510-022-00424-5

**Published:** 2022-08-26

**Authors:** Asma Ashari, Nik Mukhriz Nik Mustapha, Jonathan Jun Xian Yuen, Zhi Kuan Saw, May Nak Lau, Lew Xian, Alizae Marny Fadzlin Syed Mohamed, Rohaya Megat Abdul Wahab, Chiew Kit Yeoh, Malathi Deva Tata, Sindhu Sinnasamy

**Affiliations:** 1 Department of Family Oral Health, Faculty of Dentistry, Universiti Kebangsaan Malaysia, Kuala Lumpur, Malaysia; 2 Centre for Paediatric Dentistry and Orthodontic Studies, Universiti Teknologi MARA (UiTM), Selangor, Malaysia; 3 Department of Paediatric Dentistry and Orthodontics, University of Malaya (UM), Kuala Lumpur, Malaysia; 4 Orthodontic Specialist Unit, Klinik Pergigian Sungai Chua, Ministry of Health, Selangor, Malaysia; 5 Orthodontic Speciaist Unit, Klinik Pergigian Bandar Botanik, Ministry of Health, Selangor, Malaysia

**Keywords:** Orthodontic retainers, Dentoalveolar expansion, Relapse, Treatment outcome, Patient-centered outcome, Controlled clinical trial

## Abstract

**Objectives:**

To compare the clinical effectiveness of Hawley retainers (HRs) and modified vacuum-formed retainers (mVFRs) with palatal coverage in maintaining transverse expansion throughout a 24-month retention period and to assess the subjects’ perception toward the retainers.

**Materials and methods:**

The trial accomplished blinding only by the outcome assessor and data analyst. Data were collected from post-orthodontic treatment patients who met the inclusion criteria. Thirty-five subjects were randomly allocated using a centralized randomization technique into either mVFR (*n* = 18) or HR group (*n* = 17). Dental casts of subjects were evaluated at debond (T0), 3-month (T1), 6-month (T2), 12-month (T3), and 24-month retention (T4). The intercanine width (ICW), interpremolar width (IPMW), interfirst molar mesiobuccal cusp width (IFMW1), and interfirst molar distobuccal cusp width (IFMW2) were compared between groups over time using Mixed ANOVA. A pilot-tested and validated questionnaire consisting of six items were given at T4. Subjects were instructed to rate their retainer in terms of fitting, speech, appearance, oral hygiene, durability, and comfort on a 100-mm Visual Analogue Scale (VAS).

**Results:**

No statistically significant differences in arch width were found between the two groups at ICW (*P* = .83), IPMW (*P* = 0.63), IFMW1 (*P* = .22), and IFMW2 (*P* = .46) during the 24-month retention period. Also, no statistically significant differences were found between perception of both retainers in terms of fitting, speech, oral hygiene, durability, and comfort (*P* > .05) after 24-month wear. The appearance of mVFRs was rated significantly higher compared to HRs (*P* < .05).

**Conclusions:**

HR and mVFR have similar clinical effectiveness for retention of transverse expansion cases in a 24-month retention period. Both retainers were perceived to be equal in terms of fitting, speech, oral hygiene, durability, and comfort. Subjects in the mVFRs group found their retainers to be significantly more esthetic than those in HRs group.

## Background

The importance of a retention regime after active orthodontic treatment is undeniable. Retention is achieved via orthodontic retainers, which can be fixed or removable. Hawley retainers (HRs) have been used for over a century as an effective removable orthodontic retainer [1]. Since the advent of vacuum-formed retainers (VFRs) in 1971, it has become increasingly popular and is the more common removable retainer type prescribed in countries such as Australia, New Zealand, Ireland, Netherlands, India, and Malaysia [2–5]. They are cost effective and easier to fabricate [6].

**Fig. 1:**
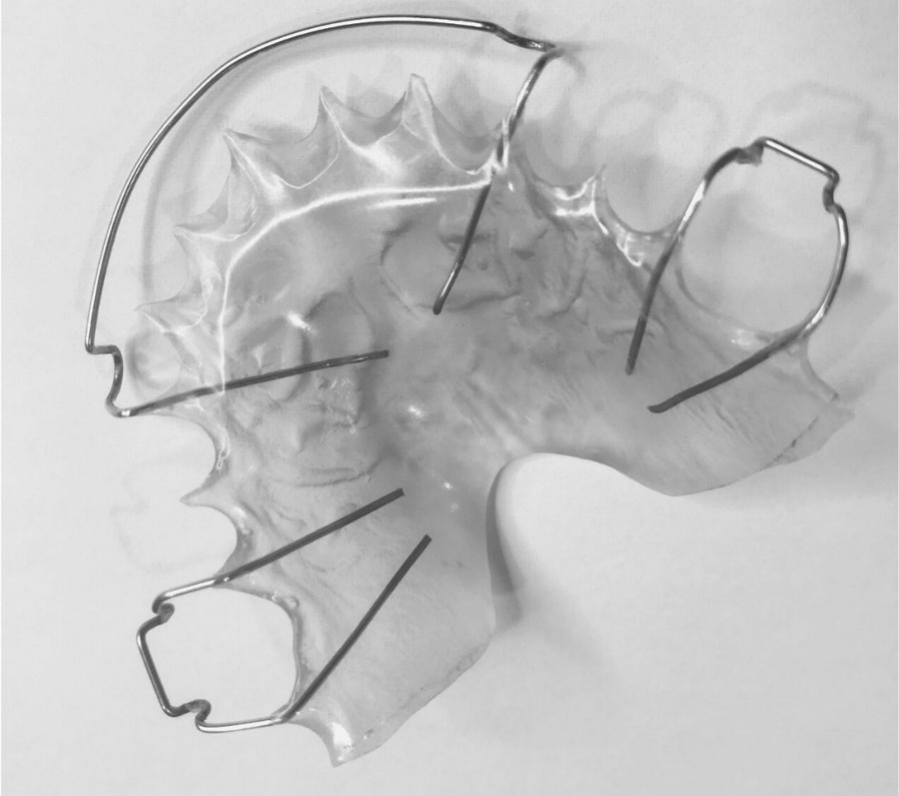
Hawley retainer used in the trial

**Fig. 2:**
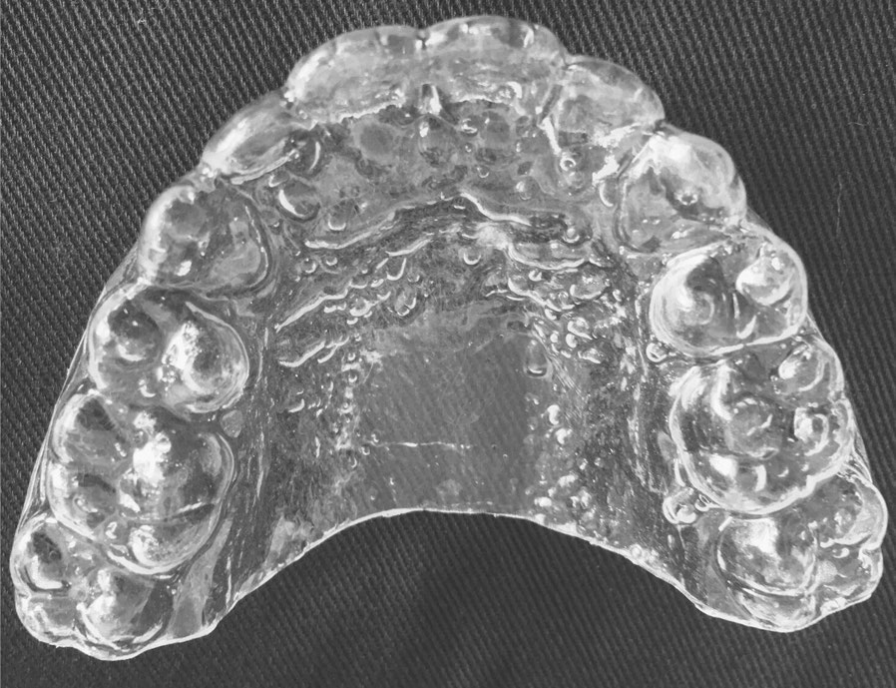
Modified vacuum-formed retainer with palatal coverage

In our previous randomized clinical trial, we compared the clinical effectiveness of the HRs (Fig. 1) and modified vacuum-formed retainers (mVFRs; Fig. 2) in retaining lateral expansion cases, by measuring arch width changes over time. Palatal coverage was added to the conventional U-shaped VFRs to impart rigidity. Our data showed that mVFRs have similar effectiveness to HRs in maintaining trans-arch stability at 6 months and 12 months following transverse expansion [7, 8]. This randomized clinical trial prospectively compares the clinical effectiveness of mVFRs and HRs in expansion cases by measuring maxillary arch width changes over a 24-month period. It is imperative to evaluate the outcome of these retention regimes over a longer period, as transverse correction usually warrants prolonged retention.

While the clinical effectiveness is being evaluated, it would be valuable to assess patients’ acceptance toward mVFRs as compliance level to removable retainers is key to retention success, which is often suboptimal [9]. Non-compliance may be related to discomfort, inconvenience, esthetics, and/or speech disturbances [6, 10]. Many advantages of VFRs, especially those related to comfort and speech, are owing to the lack of palatal coverage in VFRs [11]. While studies have been conducted to investigate patient perception of conventional VFRs, there is a lack of evidence in patient perception of VFRs with palatal coverage. Assessing and understanding patients’ perception of their retainer is of paramount importance as this information will enable clinicians to make better decisions when prescribing orthodontic retainers.

### Specific objective and hypothesis

The primary aim of the current study was to compare the clinical effectiveness of mVFRs and HRs in expansion cases by measuring maxillary arch width changes over a 24-month retention period. The secondary aim was to assess the subjects’ perception of their retainer, after either one for 2 years via a questionnaire. The null hypothesis for the primary outcome was that there was no significant difference in the effectiveness of the retainers in terms of the maintenance of transarch stability.

## Materials and methods

This RCT was approved by UKM Research and Ethics Committee (UKMPPI/111/8/JEP-2018-724), the National Medical Research and Ethics Committee (NMRR-18-3639-44877) and registered with ClinicalTrials.gov (NCT04237298). This trial was a two-arm parallel prospective multi-centre randomized controlled trial with a 1:1 allocation ratio conducted in Orthodontic Specialist Unit of Universiti Kebangsaan Malaysia (UKM), Klinik Pergigian Bandar Botanik Klang, and Klinik Pergigian Sungai Chua Kajang (the orthodontists had more than five years of experience). There were no changes to the methods after trial commencement.

### Sample size calculation

The sample size for the randomized clinical trial was calculated with reference to a previous study by Petrén and Bondemark, using a two-mean comparison with a significance level of 0.05 and an 90% power level to detect a clinically meaningful difference of 2.0 mm in arch expansion with a standard deviation (SD) of 1.5 mm [12]. For each arm, the power analysis yielded a total of 12 individuals. A 10% sample size attrition accounted for any loss to follow-up or non-compliance. A combined total of 28 participants were required, as there were two groups.

### Data collection

The initial sample was recruited between August 2019 and August 2021. Eligibility criteria included patients aged thirteen years or older at the time of debond, had existing pre-treatment dental cast, and undergone more than 3 mm of maxillary dentoalveolar expansion during treatment either with quad helix or by orthodontic archwires. Orthodontists, technicians, and researchers were trained and calibrated prior to the start of the study. On debond day, the clinician inspected all orthodontic patients and made four linear measurements: intercanine width (ICW—the distance between the canine cusp tips), interpremolar width (IPMW—the distance between the premolar cusp tips), interfirst molar width 1 (IFMW1—the distance between the mesiobuccal cusp), and interfirst molar width 2 (IFMW2—the distance between the distobuccal cusp). The measurements were made intraorally and then on the pre-treatment casts using a Tuten electronic digital calliper (CSM Engineering Hardware (M) Sdn. Bhd, MY) with a precision of 0.01 mm. The clinician repeated both the measurements once to ensure accuracy. Once the debond model was available, an independent researcher again calculated the total amount of expansion from pre-treatment and post-treatment (LX). At least two or more points were expanded (> 3 mm) to be included in the trial.

For eligible subjects, the researcher gave them an information sheet, explained the trial, followed by obtaining informed consent. The subjects were randomly assigned to one of two groups, either an upper removable HR or mVFR covering the palate. The clinician determined the type of lower retainers. Next, trained technicians standardized the design of the upper retainers. An Essix plastic sheet 0.040″ (1 mm) (Dentsply Raintree Essix, Sarasota, USA) was used for the fabrication of mVFRs, while HRs were constructed using the acrylic resin (Scheu-Dental, Iserlohn, GER) and stainless steel wire Chromium Coil 0.70 mm (Scheu-Dental, Iserlohn, GER).

Within 24 h of debonding, retainers were inserted. The subjects were told to wear the retainers full-time for the first 6 months, then only at night for the next 6 months. They were allowed to remove their retainers for cleaning, eating, and drinking. The implications of failing to comply were also discussed. Once a month, each subject received a text reminder to wear their retainers to improve compliance. Retainers were also checked during each appointment to verify that they were fitted correctly.

Impressions were taken for dental casts construction at four occasions and later were measured: at debond and retainers were fitted (T0), 3-month (T1), 6-month (T2), 12-month (T3), and 24-month (T4) retention. Alginate (Major Prodotti Dentari S.p.A., Moncalieri, ITL) and yellow stone (Samwoo Co Ltd, Ulsan, KR) were used for the impressions and dental casts, respectively. At the 24-month appointment, the subjects were given the questionnaire to evaluate their perception toward their retainer.

The primary (clinical) outcome in this study was the transarch stability measured by ICW, IPMW, and IFMW. Four linear measurements (ICW, IPMW, IFMW1, and IFMW2) were made on each cast. For each point of measurement, the average of three measurements was taken. The independent researcher (LX) used a Tuten electronic digital calliper (CSM Engineering Hardware (M) Sdn. Bhd, MY) to collect data with a precision of 0.01 mm.

The randomization sequence was computer-generated and carried out in blocks of 18. External involvement was incorporated into a centralized randomization process. In order to avoid selection bias and protect the assignment sequence until allocation, co-researchers on-site sought suitable individuals and contacted the centre by phone after patients agreed to participate. An independent researcher (KE) performed this before trial commencement, who also acted as the trial coordinator.

The researcher (LX), blinded to each subject's retention regime, measured each dental cast with an identity document. During measurement, all patient identification information on the dental casts was hidden using opaque tape. The sequence of the casts was also randomized prior to measurement. Only one dental cast was measured at a time without showing any previous measurements or assigned retainer. Clinicians, assistants, and subjects were not feasible to be blinded in this trial.

The secondary (patient-centered) outcome measures were collected through questionnaire. Subjects were given questionnaires on patient acceptance on orthodontic retainers.

### Questionnaire development

A questionnaire on patient acceptance on orthodontic retainers, originally developed by Ngan et al. [13] and modified by Saleh et al. [14], was used in this study. Following pilot testing, the questionnaire was forward and backward translated followed by three validity tests and two reliability tests, namely content validity, face validity, criterion validity, test–retest reliability, and internal consistency. It was validated with good to excellent reliability.

The questionnaire uses a Visual Analogue Scale (VAS) to ask six questions related to subject acceptance of orthodontic retainers in fitting, speech, appearance, oral hygiene, durability, and comfort. The VAS scale uses a 100 mm line, on which the opposite ends make extreme points (Very comfortable and very uncomfortable). The participants marked on this line according to the value of their response and later it was quantified using a ruler. It was measured from right to left (in mm). A mark closer to the right and thus a lower score meant the participant felt more uncomfortable and closer to the left and thus a higher score meant the participant felt more comfortable. The average of two measurements was used for each scale of measurement.

### Statistical analysis

All statistical tests were conducted using Statistical Package for Social Sciences (SPSS version 26.0; International Business Machines Corp, Armonk, N.Y.).

#### Clinical outcomes

Measurement reliability was determined via the intraclass correlation coefficient (ICC). One month after initial measurements, 20 casts were selected at random, and the intra-rater reliability test (LX) was performed. The inter-rater reliability test (LX, AA) was performed on another 20 randomly selected casts. The results (Table 1) were excellent intra-rater reliability (1.00) and inter-rater reliability (0.98).

**Table 1:** Intra-class correlation coefficient in the arch width measurements

ICC	*n*	Coefficient	95% confidence interval		*P* value
			Lower bound	Upper bound	
Intra-rater reliability	20	1.00	1.00	1.00	< .001*
Inter-rater reliability	20	0.98	0.53	1.00	< .001*

ICC indicates intra-class correlation coefficient; n, sample size; *, statistical significance

The data were determined to be normally distributed via the Shapiro–Wilk test. Thus, parametric statistics were applied. The mean arch width changes over time between retention regime groups during the follow-up period were compared using the Mixed ANOVA test. All tests were performed at a significance level of 0.05. For missing outcomes, an intention-to-treat (ITT) analysis was done by computing the mean difference between two consecutive time points. The mean difference was added to the data obtained at the time points before the missing data points or to estimate the missing outcome.

#### Patient-centered outcomes

To assess intra-rater agreement, each measurement was repeated once by the same examiner after one month and analyzed using the intraclass correlation coefficient (ICC). The data measurement had excellent intra-rater reliability with an ICC score of 0.90.

The data were reported descriptively with tables as mean and standard deviation of each group’s rating for each question. The data were found to be normally distributed via the Shapiro–Wilk test. The mean differences in age, gender and perception between retainer groups were evaluated using independent samples *t* test at a 0.05 significance level.

## Results

### Participants flow

**Fig. 3:**
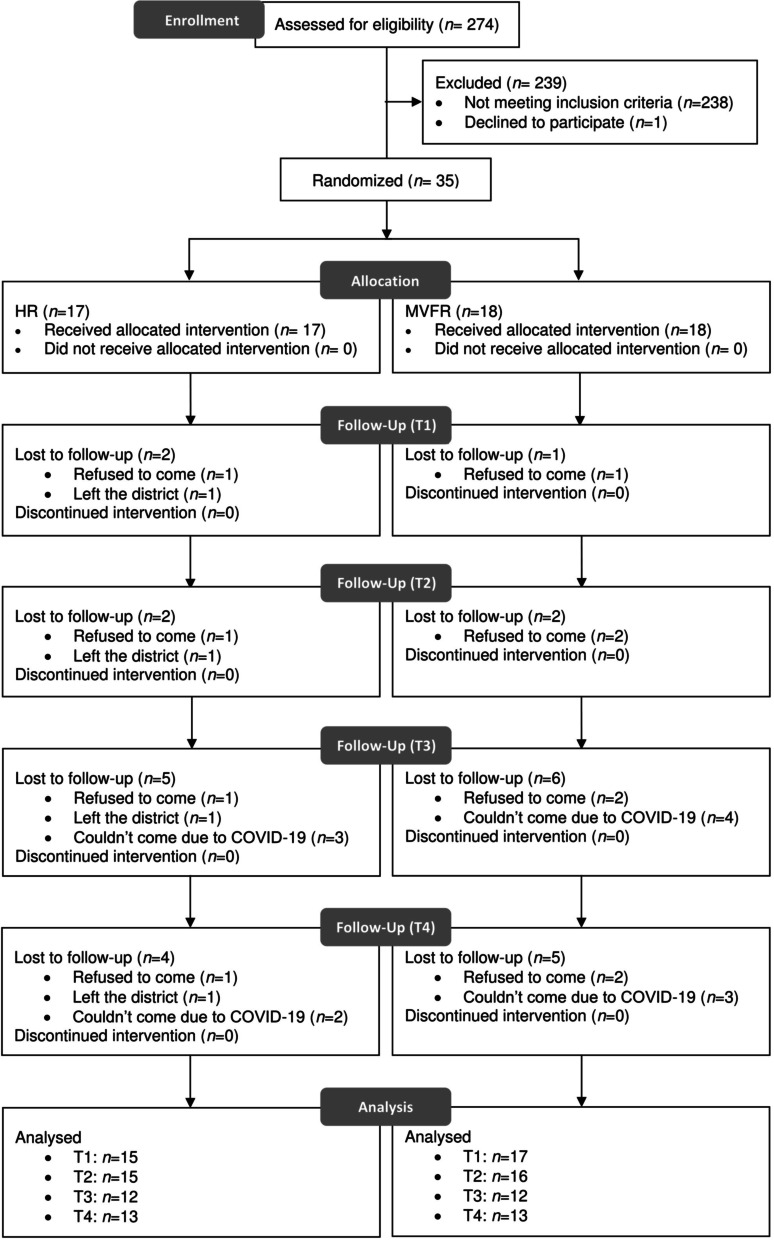
Consolidated Standards for Reporting of Trials participant flow diagram

A total of 274 patients with planned maxillary expansion were examined for inclusion in the study, with 239 of them being ineligible. The reasons for exclusion were that 225 had less than 3 mm of expansion, ten had missing pre-treatment dental cast, three was due to the clinicians deciding not to randomize the retainers, and one patient declined to participate in the study. As a result, 35 patients were chosen at random for the clinical trial. There were dropouts at various points during the analysis (Fig. 3). A total of 26 subjects agreed to participate in the questionnaire study.

### Baseline data

The age and gender of the groups were well matched, and there were no significant differences between them (*P* > 0.05; Table 2).

**Table 2:** Age and gender distribution of subjects for the groups

Variable	HR group (*n* = 17)	mVFR group (*n* = 18)	Total (*n* = 35)	*P* value
Age at debond (mean ± SD)	21.88 ± 4.12	22.06 ± 5.88	21.97 ± 5.03	.92
**Gender: *n* (%)**				.56
Male	5 (29)	7 (39)	12 (34)	
Female	12 (71)	11 (61)	23 (66)	

HR indicates Hawley retainer; mVFR, modified vacuum-formed retainer; SD, standard deviation; n, sample size

### Numbers analyzed, outcomes, and estimation

#### Clinical outcome

The mean and SDs of arch width variations in the HR and mVFR groups at five-time points are shown in Table 3. Throughout the retention period, there were no statistically significant differences (*P* > 0.05) in all width values between the two groups. In general, mean arch widths decreased across the trial period, with the exception of ICW, which rose from T3 to T4. Although the IFMW1 and IFMW2 scores for the HR group increased from T0–T1, they declined after that. Nevertheless, when comparing the arch widths at the beginning and at the end of the trial, all arch widths demonstrated a decrease. The IPMW in the mVFR group declined most between baseline (T0) and final (T4) analysis (47.10–46.31 mm).

**Table 3:** Mixed ANOVA interaction effect

Variable	Time interval	HR group	95% confidence interval	mVFR group	95% confidence interval	Mixed ANOVA *P* value (Interaction effect)
		Mean ± SD (mm)		Mean ± SD (mm)		
ICW						.83
	T0	37.20 ± 2.46	36.09–38.30	38.04 ± 2.00	36.97–39.12	
	T1	37.19 ± 2.55	36.05–38.32	37.93 ± 2.02	36.83–39.03	
	T2	37.07 ± 2.53	35.96–38.17	37.80 ± 1.92	36.73–38.87	
	T3	36.80 ± 2.78	35.61–37.99	37.55 ± 1.99	36.40–38.70	
	T4	36.90 ± 2.65	35.76–38.04	37.65 ± 1.93	36.54–38.76	
IPMW						.63
	T0	45.39 ± 1.88	44.33–46.45	47.10 ± 1.84	46.04–48.17	
	T1	45.37 ± 1.88	44.29–46.46	46.87 ± 1.92	45.79–47.96	
	T2	45.26 ± 1.94	44.10–46.41	46.77 ± 2.08	45.62–47.92	
	T3	44.80 ± 1.92	43.62–45.97	46.40 ± 2.17	45.22–47.57	
	T4	44.61 ± 2.13	43.39–45.82	46.31 ± 2.12	45.09–47.52	
IFMW1						.22
	T0	51.16 ± 1.73	50.12–52.21	53.35 ± 2.29	52.37–54.33	
	T1	51.31 ± 1.66	50.27–52.36	53.15 ± 2.35	52.17–54.14	
	T2	51.16 ± 1.79	50.07–52.26	53.01 ± 2.43	51.97–54.04	
	T3	50.88 ± 1.82	49.78–51.98	52.93 ± 2.42	51.89–53.97	
	T4	50.74 ± 2.00	49.64–51.85	52.86 ± 2.31	51.82–53.90	
IFMW2						.46
	T0	52.34 ± 2.09	51.14–53.54	54.26 ± 2.56	53.13–55.39	
	T1	52.50 ± 2.16	51.24–53.75	54.20 ± 2.71	53.02–55.38	
	T2	52.27 ± 2.15	51.00–53.54	54.11 ± 2.75	52.91–55.30	
	T3	52.09 ± 2.29	50.80–53.39	53.96 ± 2.75	52.73–55.18	
	T4	52.04 ± 2.21	50.78–53.31	54.05 ± 2.71	52.86–55.25	

HR indicates Hawley retainer; mVFR, modified vacuum-formed retainer; SD, standard deviation; ICW, intercanine width; IPMW, interpremolar width; IFMW1, interfirst molar mesiobuccal cusp width-; IFMW2, interfirst molar distobuccal cusp width; T0, at debond; T1, 3-month retention; T2, 6-month retention; T3, 12-month retention; T4, 24-month retention

Loss (6%—HR only) and breakage (6%—HR; 22%—mVFR) were the leading causes of retainer failure. As soon as feasible, subjects were issued new retainers with the same design.

#### Patient-centered outcome

Table 4 summarizes the comparison of perception to retainers between subjects from both groups based on the VAS questionnaire data. There were no significant differences (*P* > 0.05) between the two groups for all the variables except for appearance (*P* < 0.05).

**Table 4:** Independent samples T-test

Variable	Type of retainer	Mean	Mean difference	95% CI of the difference	*P* value
Fitting	mVFRs	7.08	1.24	− 0.526, 3.013	.160
	HRs	5.84			
Speech	mVFRs	7.32	1.76	− 0.247, 3.759	.083
	HRs	5.56			
Appearance	mVFRs	8.02	1.84	0.209, 3.460	.029*
	HRs	6.18			
Oral hygiene	mVFRs	7.63	0.77	− 1.053, 2.606	.389
	HRs	6.86			
Durability	mVFRs	7.05	0.25	− 1.700, 2.192	.797
	HRs	6.80			
Comfort	mVFRs	7.69	1.63	− 0.261, 3.521	.088
	HRs	6.06			
Total mean additive score	mVFRs	7.46	1.24	− 0.277, 2.772	.104
	HRs	6.22			

mVFRs indicate modified vacuum-formed retainers; HRs, Hawley retainers; CI, confidence interval

Level of significance set at 0.05. *Significant *P* values

## Discussion

### Findings and interpretations

The current trial was expanded to explore the clinical and patient-centered outcome of HR and mVFR in sustaining maxillary arch expansion for up to 24 months [7, 8]. ICW, IPMW, and IFMW were chosen as clinical outcome measures to reflect transarch stability, demonstrating the clinical effectiveness of retention techniques in preventing relapse, as previously demonstrated in numerous studies [15–19]. 100-mm VAS with six questions were used to evaluate patient-centered outcome, i.e., subjects’ perception on these orthodontic retainers.

Subjects from both retainer groups had mean ages of 22.58 years and 21.07 years for mVFRs and HRs, respectively. There were more females subjects compared to males in the trial, which is a frequent phenomenon in studies investigating orthodontic appliances [14, 19–23].

#### Clinical outcome

The average expansion for all measurement points was 4.35 ± 2.40 (ICW), 4.67 ± 1.74 (IPMW), and 3.05 ± 3.59 (IFMW). For the largest value among the measurement points, there was an average of 6.05 ± 2.73 mm arch width. Buccal inclinations of teeth, bone remodeling, and reduced bone thickness, notably in the buccal aspect, have all been described during dentoalveolar extension [16, 24]. The changes that occur after treatment have been attributed to relapse following orthodontic expansion [25–28], as well as growth changes. These differences were neither statistically or clinically significant between groups or time points from the 24-month post-retention period (Table 3). When the differences of the time points were calculated for ICW, IPMW, and IFMW (Table 5), changes occurred across the trial period with values below 1 mm, independent of the retention regime. A three-year follow-up of a randomized clinical trial on dentoalveolar expansion in the mixed dentition revealed a relapse of less than 1 mm [29], given that expansion is normally more stable in growing children.

**Table 5:** Mean difference (in mm) between the time points

Variable	HR	mVFR
*ICW*		
T1–T0	− 0.01	− 0.11
T2–T1	− 0.12	− 0.13
T3–T2	− 0.27	− 0.25
T4–T3	0.10	0.10
T4–T0	− 0.30	− 0.39
*IPMW*		
T1–T0	− 0.01	− 0.23
T2–T1	− 0.12	− 0.10
T3–T2	− 0.27	− 0.37
T4–T3	0.10	− 0.09
T4–T0	− 0.30	− 0.79
*IFMW1*		
T1–T0	− 0.01	− 0.20
T2–T1	− 0.12	− 0.14
T3–T2	− 0.27	− 0.08
T4–T3	0.10	− 0.07
T4–T0	− 0.30	− 0.49
*IFMW2*		
T1–T0	− 0.01	− 0.06
T2–T1	− 0.12	− 0.09
T3–T2	− 0.27	− 0.15
T4–T3	0.10	0.09
T4–T0	− 0.30	− 0.21

HR indicates Hawley retainer; mVFR, modified vacuum-formed retainer; ICW, intercanine width; IPMW, interpremolar width; IFMW1, interfirst molar mesiobuccal cusp width-; IFMW2, interfirst molar distobuccal cusp width; T0, at debond; T1, 3-month retention; T2, 6-month retention; T3, 12-month retention; T4, 24-month retention

Conversely, another study on relapse after dentoalveolar expansion in teenage patients discovered more than 1 mm of relapse over a year [16]. The researchers concluded that this could be due to compliance issues, where subjects were sent text reminders monthly in the present trial. It is worth noting that, except for IFMW2, the HR group did exceptionally well from T1–T0 and for the total difference T4–T0 in the current study, despite the fact that these results were not statistically or clinically significant.

Over a 24-month retention period, the main outcomes of the current trial revealed no statistically significant differences between HR and mVFR in all mean arch width changes. This finding is comparable with previous investigations which compared HR's stability and the conventional VFR without palatal coverage in non-expansion cases [17–19, 30, 31]. The findings showed that the HR and the mVFR are equally effective in sustaining maxillary transverse expansion after 24 months. The mVFR's extended palatal coverage combined with the rigidity of the thermoplastic material [18] may have improved their physical qualities, allowing them to maintain an expanded arch akin to HRs, which have always been regarded as more rigid and better for transarch stability [26, 32]. Another reason for the mVFR's effectiveness could be the three-dimensional coverage of the teeth, including palatal coverage, which, in principle, would better preserve dental inclination changes over HR [33]. The findings of this study suggest that mVFRs would be a suitable option for expansion cases because it is easier to produce and does not necessitate any additional technical abilities. However, the mVFR group reported more retainer breakages than the HR group, with no further breakages after the one-year trial (6%—HR; 22%—mVFR) [7].

#### Patient-centered outcome

Subjects perceived the mVFRs as significantly more esthetically pleasing compared to HR. This finding is in agreement with the results of multiple studies and a systematic review [6, 14, 18, 34]. Several authors suggested that this was attributed to the transparent nature of VFRs as compared to metal showing in HRs [6, 34]. In addition, Hichens et al. found that VFRs caused less embarrassment when worn in public compared to HRs [6]. The superior esthetics might be a factor for the increasing popularity of VFRs [2, 4, 6, 35, 36]. Nevertheless, Pratt et al. reported no differences in regard to the appearance of HRs and VFRs [37].

In the present study, no significant differences were found between the perception of the two retainers in terms of speech. This is inconsistent with multiple studies that found VFRs cause less disruption in speech [6, 38, 39]. Using acoustic analysis, both Wan et al. and Atik et al. found that the change in articulation was more obvious in patients wearing HRs compared to conventional VFRs [38, 39]. In the present study, the VFRs were modified with palatal coverage, therefore imparts greater speech disturbances compared to conventional horseshoe-shaped VFRs. As evidenced by Stratton and Burkland, retainers with palatal coverage tend to result in greater speech disturbances compared to those without palatal coverage [11]. This may explain the insignificant differences in perception of speech disruption between mVFRs and HRs.

The results also found no significant differences in perception of comfort between both types of retainers. In the literature, VFRs without palatal coverage demonstrated superior comfort compared to retainers with palatal coverage [14, 40]. The mVFRs used in the present study had palatal coverage similar to HR, which could explain the insignificant differences. However, Hichens et al. found no difference in comfort level associated with VFRs and HRs, despite the VFRs used in their study did not have palatal coverage [6].

The subjects also reported no difference in perceived durability between the two retainers. This is inconsistent with the findings of Saleh et al. who found subjects perceived HRs to be significantly more durable [14]. The inherent flexibility of the traditional horseshoe-shaped VFRs might come across as less durable to subjects [14]. In the previous study, it is postulated that even though the material is in theory not as rigid as the acrylic in Hawley, the palatal coverage had increased the strength of mVFRs [7]. This increased strength and reduced flexibility of the mVFRs might have been the reason for equal perception of durability between both the retainers. There was conflicting evidence in the survival times of the two retainers, possibly due to the varying thickness, material, design (amount of gingival coverage) of VFRs and the inconsistency in individual patient care and habits, e.g., bruxing. Note that these studies used VFRs without palatal coverage [6, 14, 41]. However, our trial reported a higher number of breakages in the mVFRs group compared to the HRs group (6%—HRs; 22%—mVFRs) within one year of retainer wear, which did not increase after the first year [7].

There was also no significant difference between the fitting and oral hygiene perception of HRs and mVFRs. This is consistent with result from a randomized trial conducted by Saleh et al. where they compared the fitting and oral hygiene perception between HRs and VFRs without palatal coverage [14].

The results for patient-centered outcomes suggest that mVFRs are comparable with HRs in aspects of fitting, speech, oral hygiene, durability, and comfort, with mVFRs being superior in terms of appearance.

### Limitations

By the time point of analysis, the relative dropout rates had risen (Fig. 3). Since January 2020, the main reason has been the COVID-19 pandemic [42]. However, one subject in each HR and mVFR group refused to come due to COVID-19 concerns returned for their T4 visit when the situation improved, which increased the total number of subjects by two at the end of the trial. An ITT analysis was used to reduce the possibility of bias generated by comparing groups with different prognostic variables.

The compliance of retainer wear was not objectively measured in the study. However, monthly text reminders were sent, and the retainers were ensured to be well fitted at each T visit to mimic the real clinical scenario. The Hawthorne effect, which may change a certain aspect of the individuals’ behavior in reaction to the reminder in this trial, remains challenging to minimize. It has been demonstrated that compliance is most substantial during the early stages, where patient participation tends to fade over time [43].

This trial was conducted on subjects who have been wearing retainers for two years and may not represent patients in other phases of retention. Since the average amount of dentoalveolar expansion was minimal, the results of the study would not be applicable to other modalities of expansion such as skeletal expansion with RME or SARPE.

### Recommendations

Long-term retention phase may harm teeth and gingival health. In addition, even the long-term wear of VFRs has demonstrated a significant premature occlusal contact in the posterior teeth and an anterior open bite [44, 45]. All these possible effects could be evaluated in future studies. Furthermore, the authors suggest that the questionnaire used in this study could be used in future studies investigating the patient-centered outcomes of various orthodontic retainers since this is the only validated questionnaire on patient acceptance of orthodontic retainers.

## Conclusions

- HRs and mVFRs have similar clinical effectiveness for retention of transverse expansion over a 24-month retention period
- No subjective differences between mVFRs and HRs in terms of fitting, speech, oral hygiene, durability, and comfort were observed.
- mVFRs were perceived to be significantly more esthetic than HRs.

## Data Availability

The datasets used and/or analyzed during the current study are available from the corresponding author on reasonable request.

## Abbreviations

HRs: Hawley retainers
mVFRs: Modified vacuum-formed retainers
ICW: Intercanine width
IPMW: Interpremolar width
IFMW1: Interfirst molar mesiobuccal cusp width
IFMW2: Interfirst molar distobuccal cusp width
T0: Dental casts evaluated at debond
T1: Dental casts evaluated 3-month of retention
T2: Dental casts evaluated 6-month of retention
T3: Dental casts evaluated 12-month of retention
T4: Dental casts evaluated 24-month of retention
VAS: Visual analogue scale
ICC: Intra-class correlation coefficient
